# Relationship Among Physical Literacy, Mental Health, and Resilience in College Students

**DOI:** 10.3389/fpsyt.2021.767804

**Published:** 2021-12-13

**Authors:** Ruisi Ma, Ting Liu, Kim Wai Raymond Sum, Tianyu Gao, Minghui Li, Siu Ming Choi, Yan Huang, Wenyi Xiang

**Affiliations:** ^1^School of Physical Education, Jinan University, Guangzhou, China; ^2^Department of Sports Science and Physical Education, Faculty of Education, The Chinese University of Hong Kong, Shatin, Hong Kong SAR, China; ^3^The Nethersole School of Nursing, Faculty of Medicine, The Chinese University of Hong Kong, Shatin, Hong Kong SAR, China

**Keywords:** physical literacy, mental health, resilience, mediation, association, relationship

## Abstract

**Objectives:** The objective of the study is to examine the relationship among physical literacy, mental health, and resilience in college students.

**Methods:** The study is a cross-sectional study. Participants (*N* = 5,265; 46.6% males) completed perceived physical literacy instruments, mental health continuum short forms, and the 12-item child and youth resilience measures. Mediation models were applied to explore the association among three concepts.

**Results:** Physical literacy, resilience, and mental health were significantly linked with each other. In the mediation model, the direct effect of physical literacy on mental health was 0.49. The indirect effect of physical literacy on mental health through the mediation of resilience was 0.97. The mediating effect of resilience accounts for 66.30% of the total effect, indicating the partial mediating effect of resilience in the relationship between physical literacy and mental health. In more detailed models, resilience was found to mediate the relationship between physical literacy and social and psychological wellbeing, accounting for 61.02% and 56.92% of the total effect, respectively. In addition, resilience acted as full mediator in the relationship between physical literacy and emotional wellbeing (>100%). These findings suggest that physical literacy increases mental health by improving resilience.

**Conclusions:** This is the first time to connect physical literacy with mind factors. The mediating effect of resilience on the relationship between physical literacy and mental health was found. Our findings support the development of physical literacy in universities as part of a holistic approach to supporting the wellbeing and mental health of undergraduates. This study provides a new perspective for the development of large-scale interventions in the health of body and mind in college students.

## Introduction

Physical literacy is a multidimensional concept that includes physical, affective, and cognitive factors and is defined as the motivation, confidence, physical competence, knowledge, and understanding to value and take responsibility for engagement in physical activities for life (1, 2). The concept roots in existential and phenomenological philosophy, which sees physical literacy as an essential component in human thriving and a construct of embodiment to quest harmony and unity among mental, physical, and environmental states (1, 3). Thus, a growing body of research has suggested that physical literacy is the foundation of lifetime physical activity participation (4–7). Previous studies demonstrate the positive relationships between physical literacy and physical activity levels (8, 9). Evidence also supported the beneficial effects of physical literacy on physical fitness (8, 10). Most studies have focused on linking physical literacy with the physical domain, expecting the positive impact on approaches to promote participation in physical activities through physical literacy (3). However, the embodiment of physical literacy supports the belief that body and mind cannot be separated (3). Expecting that physical and mental factors are equally linked with physical literacy is reasonable. Nevertheless, as an integral aspect of physical literacy, the mental health factor has not yet captured attention in this area.

## Literature Review

### Physical Literacy and Mental Health

The World Health Organization (WHO) defined mental health as “a state of well-being in which every individual realizes his or her own potential, can cope with the normal stresses of life, can work productively and fruitfully, and is able to make a contribution to her or his community” (11). Three key components were included in this definition: well-being, effective functioning in individual life, and effective functioning in community life, and the definition builds on two longstanding traditions in studies on life going well (12, 13): the *hedonic* tradition focus on feelings of happiness (emotional wellbeing), whereas the *eudaimonic* tradition emphasizes optimal functioning in individual and social life (psychological and social wellbeing) (14, 15). Mental health is vital to overall well-being, which is just as important as physical health (16). Furthermore, positive mental health is more than the absence of mental disorders or disabilities. Mental health is a state of well-being in which individuals are able to think, emote, communicate, earn a living, and enjoy the ability to live (12). It is a state that needs to be promoted and protected over time.

Based on the model proposed by Whitehead, physical literacy was developed from three domains: affective, physical, and cognitive. At the macro level, physical literacy emphasizes the inseparability of body and mind, with several dimensions interacting with each other. At the micro level, physical literacy emphasizes lifelong movement and positive attitudes (3). Meanwhile, mental health is considered to be the holistic triad of cognitive, behavioral, and affective wellbeing (17). In this respect, it shares partial commonalities with physical literacy (18). The influence of environmental factors on mental health has been demonstrated (19). Similarly, the effects of how environmental factors impact physical literacy were also examined (5). Such common characteristics, as well as the commonality in improving the wellbeing and quality of life of individuals, could be the basis for the association between physical literacy and mental health. However, yet, to date, empirical evidence on the association between physical literacy and psychological or mental health factors remains scarce. One recent study among 184 early adolescents found that physical literacy was positively associated with positive emotions and negatively associated with negative affect (20). Wang et al. used longitudinal studies to demonstrate the interaction between physical literacy and psychological satisfaction among 549 University students (21). However, modern mental health is not only related to positive mood (emotional wellbeing), but it also includes the presence of positive functioning in individual life (psychological wellbeing) and community life (social wellbeing). Moreover, the mechanisms underlying these positive outcomes have yet to be identified. Therefore, there is a need for a more comprehensive study of the relationship between physical literacy and mental health (including emotional, psychological, and social wellbeing), and the contribution of the mediating factors involved in this relationship.

### Physical Literacy, Mental Health, and Resilience

Resilience is a multifaceted concept that is defined as the ability of a dynamic system to adapt to the interference that threatens system function, viability, and development (22, 23).

Resilience comes from quality interaction with the environment, which constantly promotes or maintains positive emotions and eventually achieves physical and psychological harmony (24). Thus, the resources that the environment provided influenced the development or maintenance of optimal mental, social, and physical health of youth. Meanwhile, the concept of resilience advocates the development of the ability of people to grow in adversity through quality interaction with the environment and to enjoy the resources provided by it. This concept is consistent with the concept of physical literacy that claims interaction with the surroundings to improve physical and social settings (22, 25). Furthermore, core elements of both physical literacy and resilience are enhanced when an environment is established that helps develop the ability to overcome challenges, obstacles, or adversity. In resilience, this process suggests that appropriate exposure to adversity in proper settings can help individuals gain coping experiences and strategies, which will provide advantages in future encounters (26). Similarly, in physical literacy, engaging in appropriately constructed challenging sports not only boosts confidence but also increases motivation and willingness to further participate in physical activity (3). Therefore, resilience and physical literacy both are dynamic concepts that are influenced by their environment and multidimensional factors throughout life (3, 26). Yet, to the best of our knowledge, only one study examined the association between physical literacy and resilience among 227 school children (9–12 years old) (23). Thus, further examining the relationship between physical literacy and resilience among college students is a paramount need to strengthen the power of current evidence in this area. In addition, studies have asserted that young people with high resilience can adapt quickly when they were exposed to adversity (27). Previous studies, thus, examined how resilience-based interventions can benefit the behavior, mental health, and overall wellbeing of the individual (28–32). Given the relevance of mental health to the environment (33), it is reasonable to infer that resilience, namely, the ability to bounce back, or recover, in the face of adversity, could promote mental health (34). Moreover, resilience can always serve as mediator between mental health and other mental health-related factors, such as positive affect, social support, perceived stress and risk, and coping (35–38). Therefore, it could be assumed that resilience may mediate the relationship between physical literacy and positive mental health.

Physical literacy, mental health, and resilience are correlated to some degree. The concept of physical literacy as a link between body and mind is theoretically influential in promoting mental health. Resilience, as the ability to combat adversity, should also play an active role in the ability of the individual to achieve mental health. University students are in the last stage of the education process (39). During this period, young people need to take on pressure from a changing environment and adapt to a new phase of socialization and study mode. Research has revealed concerning rates of psychological illness, such as anxiety and depression, among University students (40). Interventions based on cognitive, behavioral, and mindfulness have shown to be effective in reducing stress in University students (40). Therefore, understanding how physical and psychological domains work together can help us better appreciate the mechanisms by which the body and mind operate, and can thereby better inform the instruction of the interventions, such as physical education courses and other movement-based programs. Such a link also supports physical and psychological harmony among students, which leads to a greater sense of well-being (23). Thus, this study provided a new perspective on physical literacy, clarifying the relationship among physical literacy, mental health, and resilience among undergraduates. The hypothesis of this study are as follows:

Hypothesis 1: Physical literacy will positively influence mental health.
Hypothesis 2: Physical literacy will positively influence resilience.
Hypothesis 3: Resilience will mediate the relationship between physical literacy and mental health.

## Method

### Design and Participants

Cross-sectional data was extracted from a 4-year longitudinal study, which tracked changes in physical literacy over the life of an undergraduate under natural circumstances. The study was conducted at Jinan University, China. Questionnaires were distributed through an online website. A total of 5,835 undergraduates participated in the study, and 5,265 completed the questionnaires. The response rate was 90.23%. All participants were fully informed of the details of the study and free to withdraw from participating at any time during the process, either temporarily or permanently. The ethical approval was obtained from the IRB of Jinan University (JNUKY-2021-008).

### Measures

Physical literacy was assessed by the simplified Chinese version of perceived physical literacy instrument (PPLI-SC) (41), which is an eight-item instrument to measure the physical literacy of Chinese undergraduates. It consists of three dimensions, namely, *motivation, confidence and physical competence*, and *interaction with the environment*. Specifically, *motivation* examined whether individuals would maintain positive attitudes toward physical activity throughout their life. *Confidence and physical competence* detected whether people could move with confidence and poise in a variety of challenging situations. *Interaction with the environment* monitored whether individuals can interact with the environment in the context of each day (1). All three dimensions were defined as the core stage of Whitehead's concept of physical literacy. Each item was rated on a five-point Likert scale, ranging from strongly agree to strongly disagree. PPLI-SC was proven to be a reliable and valid instrument to measure physical literacy of Chinese undergraduates through Cronbach's alpha (α = 0.86) and confirmatory factor analysis (CFA) (factor loadings ranged from 0.60 to 0.92, RMSEA = 0.03, AGFI = 0.96, NFI = 0.97, CFI = 0.99) (41). In this study, the Cronbach's alpha was 0.91.

The simplified Chinese version of the Mental Health Continuum Short Form (MHC-SF) was translated from the MHC-SF and was used to measure positive mental health (42). The tool comprises 14 items, representing three dimensions of well-being, which are emotional wellbeing, psychological wellbeing, and social wellbeing. Emotional wellbeing represents positive affect and life satisfaction. Psychological wellbeing accesses individual functioning, including self-esteem, coping strategies, and general self-efficacy. Social well-being reveals the involvement in society, such as social participation and sense of community. The MHC-SF has shown good psychometric properties in Chinese adults through Cronbach's alpha (α = 0.92) and CFA (RMSEA = 0.08, AGFI = 0.90, NFI = 0.95, CFI = 0.95) (42). In this study, the Cronbach's alpha was 0.97.

The resilience levels of the students were measured by the simplified Chinese version of the 12-item child and youth resilience measure (CYRM-SC) (43). The CYRM is used to indicate the psychological resilience of an individual, meaning the extent to which people can use the environmental resources to thrive in adversity (44). The CYRM-SC was validated by using exploratory factor analysis and CFA, which resulted in the one-factor solution (α = 0.92, RMSEA = 0.06, CFI = 0.96, IFI = 0.96, NFI = 0.95) (43). In this study, the Cronbach's alpha was 0.93.

### Statistical Analysis

IBM SPSS 26 and PROCESS macro 3.5 were used for data analysis (45). Descriptive statistics was used to describe the characteristics of the participants. Before the analysis, normality, linearity, and homoscedasticity were examined and found to be supported. This study had two stages. First, standard regression and the bootstrap method were used to identify the mediational hypothesis. The steps were as follows: (1) Physical literacy was significantly associated with mental health. (2) Physical literacy was significantly associated with resilience. (3) Resilience was significantly associated with mental health. (4) If the boot confidence interval (CI) of the indirect effect did not contain zero, the mediating effect would be significant. Second, the mediating effect of resilience on each of the three dimensions of mental health was examined separately. In addition, previous studies have been interested in whether there are differences between men and women in the development of physical literacy (1). Thus, in this study, separate regression analyses for gender were also conducted.

## Results

A total of 5,265 current college students participated in the study. Males and females were approximately equal [male = 2,453 (46.60%); female = 2,812 (53.40%)]. The age of the students ranged between 17 and 21 years (total_age_: *M* = 18.98, *SD* = 1.10; Male_age_: *M* = 19.51, *SD* = 0.88; female_age_: *M* = 18.67, *SD* = 1.09), and most of them were 19 years old (38.40%). About half of the participants were year 1 students (*N* = 2,712, 51.50%), and others were year 2 (*N* = 2,553, 48.50%). The study streams of students were mainly in liberal arts (*N* = 1,843, 35.00%) and science (*N* = 2,685, 51.00%), followed by medicine (*N* = 527, 10.01%), and law (*N* = 210, 3.99%).

**Figure 1:**
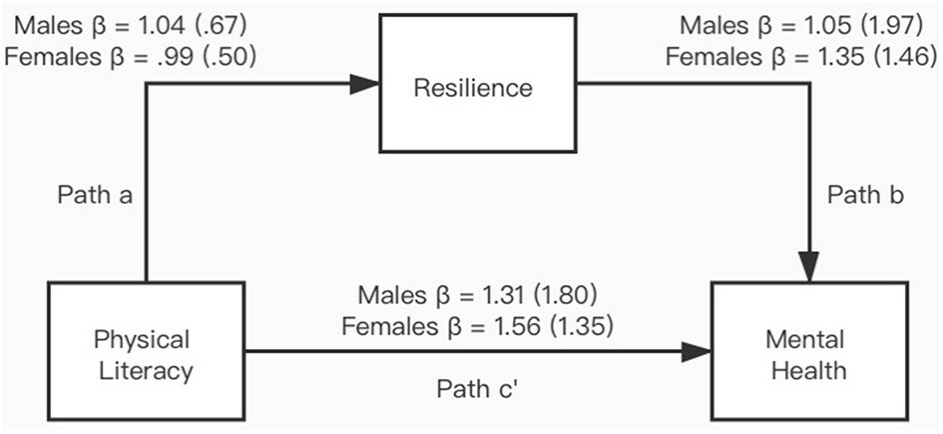
Graphical representation of the mediation model of resilience for physical literacy and mental health.

Standard linear regression was used to assess the association among physical literacy, mental health, and resilience (Table 1). All correlations were positive and strong, indicating a significant relationship among them. The three regression models were mental health = 5.63 + 1.46 × physical literacy, resilience = 17.84 + 1.01 × physical literacy, and mental health = −9.19 + 1.22 × resilience. Figure 1 presents the graphical representation of the mediation model and the regression coefficients. Association between physical literacy and mental health (Path c′) was found in males (*p* < 0.001) and females (*p* < 0.001). The connection between physical literacy and resilience (Path a), and between resilience and mental health (Path b), showed significance in each gender (*p* < 0.001). The bootstrap method was utilized to assess the mediating effect of resilience on the relationship between physical literacy and mental health (Table 2). The mediation model showed a non-zero boot CI (0.88, 1.06) with 0.49 direct effect and 0.97 indirect effect of physical literacy on mental health. Specifically, the mediating effect of resilience accounts for 66.30% of the total effect, indicating a partial mediator in the relationship between physical literacy and mental health.

**Table 1:** Results of the standard linear regression analysis among physical literacy, mental health, and resilience.

	**β (*SE*)**	***F (df)***	**(95% CI)**	***R***	**Δ*R*^2^**
All					
Physical literacy					
Mental health	1.46 (1.08)^a^	1,731.10 (1, 5,263)	(3.51, 7.76)	0.50	0.25
Resilience	1.01 (0.40)^a^	6,034.55 (1, 5,263)	(17.06, 18.63)	0.73	0.53
Resilience					
Mental health	1.22 (1.18) ^a^	2,583.95 (1, 5,263)	(−11.50, −6.88)	0.57	0.33
Males					
Physical literacy					
Mental health	1.31 (1.80)^a^	510.07 (1, 2,451)	(6.20, 13.27)	0.46	0.21
Resilience	1.04 (0.67)^a^	2,315.28 (1, 2,451)	(15.64, 18.26)	0.74	0.55
Resilience					
Mental health	1.05 (1.97)^a^	679.52 (1, 2,451)	(−4.80, 2.93)	0.51	0.26
Females					
Physical literacy					
Mental health	1.56 (1.35)^a^	1,267.31 (1, 2,810)	(0.07, 5.37)	0.53	0.28
Resilience	0.99 (0.50)^a^	3,700.63 (1, 2,810)	(17.49, 19.45)	0.73	0.53
Resilience					
Mental health	1.35 (1.46)^a^	2,067.74 (1, 2,810)	(−18.26, −12.55)	0.62	0.38

a *Correlation is significant at the 0.01 level (two tailed)*.

**Table 2:** Mediating effect of resilience on the relationship among different variables.

**Mediating effect of resilience on the model**	**Direct effect**			**Indirect effect**			**Mediating effect**		
	**All**	**Males**	**Females**	**All**	**Males**	**Females**	**All**	**Males**	**Females**
Physical literacy and mental health	0.49	0.51	0.47	0.97	0.81	1.1	66.44%^a^	61.36%^a^	70.06%^a^
Physical literacy and emotional wellbeing	−0.03	−0.05	−0.01	0.24	0.21	0.27	>100%^a^	>100%^a^	>100%^a^
Physical literacy and social wellbeing	0.23	0.30	0.19	0.36	0.32	0.38	61.02%^a^	51.51%^a^	66.67%^a^
Physical literacy and psychological wellbeing	0.28	0.26	0.30	0.37	0.28	0.43	56.92%^a^	51.85%^a^	58.90%^a^

a *The mediating effect is significant with non-zero boot CI*.

**Figure 2:**
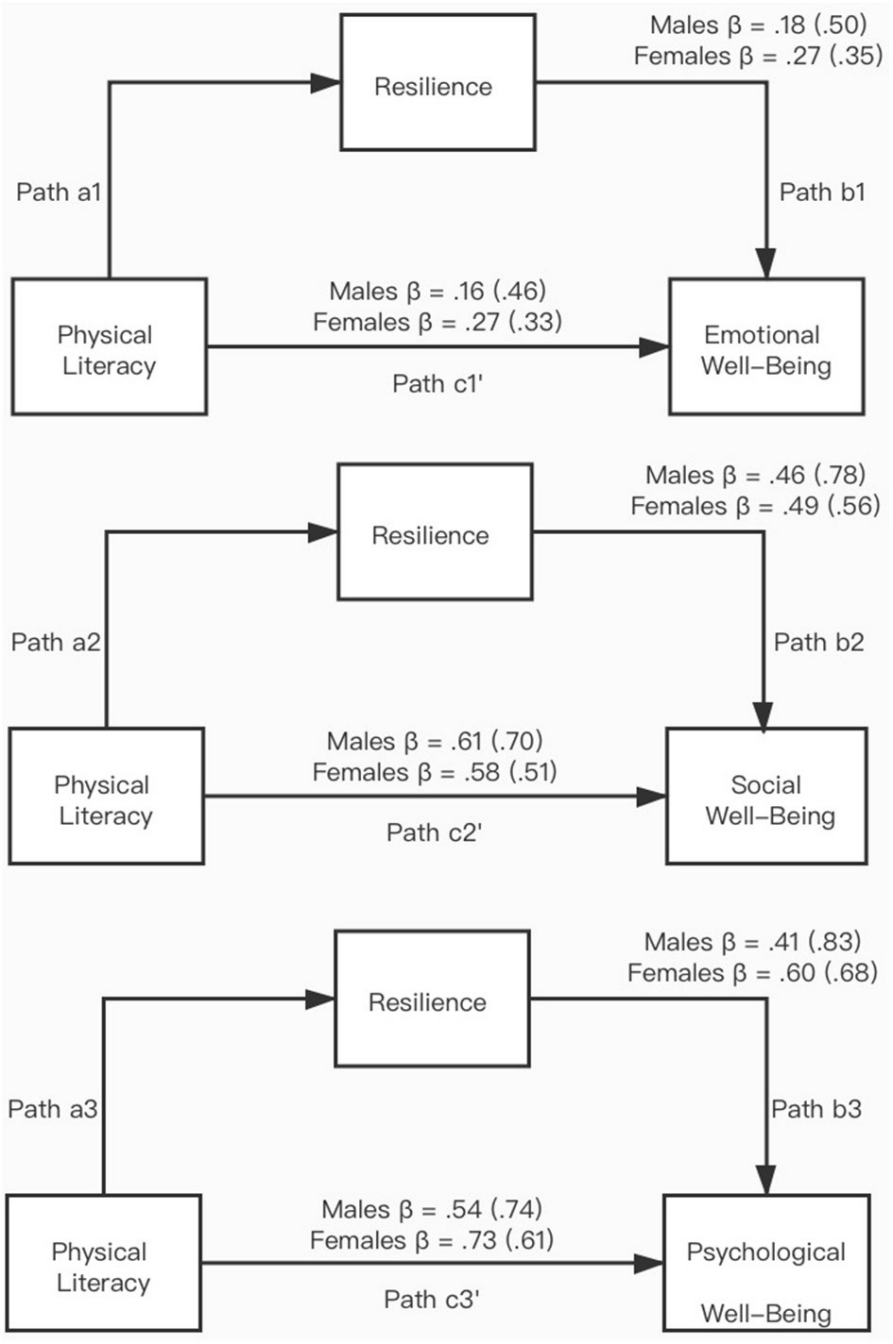
Graphical representation of the mediation model of resilience for physical literacy and the three well-being dimensions of mental health.

The bivariate Pearson's product–moment correlation coefficient (*r*) was calculated to assess the size and direction of the linear relationship among physical literacy, resilience, and the three wellbeing dimensions of mental health (Table 3). The result shows that each wellbeing dimension was significantly correlated to physical literacy and resilience. Table 2 also shows the mediating effects of resilience on the relationship between physical literacy and the three wellbeing dimensions of mental health. The mediating effect of each gender was examined as well. Same with the mediating effect of resilience on physical literacy and mental health, the model that contains social wellbeing and psychological wellbeing showed resilience as the significant partial mediator (social wellbeing: 61.02%; psychological wellbeing: 56.92%) in the mediation model. Different from the above, the mediation model pointing to emotional wellbeing showed that resilience was the significant full mediation. Specifically, with a >100% mediating effect in males and females, the relationship between physical literacy and emotional wellbeing must first pass through resilience. Without resilience, such association disappears. Figure 2 presents the graphical representation of the three mediation models and the regression coefficients.

**Table 3:** Correlations among physical literacy (PL), resilience, and mental health domains.

**Measure**	**PL**	**Resilience**	**Emotional wellbeing**	**Social wellbeing**	**Psychological wellbeing**
PL	—	0.73^a^	0.32^a^	0.51^a^	0.51^a^
Resilience	—	—	0.47^a^	0.57^a^	0.55^a^

a *Correlation is significant at the 0.01 level (two tailed)*.

## Discussion

The results of this study indicated that physical literacy, mental health, and resilience were significantly related to each other. The mediating effect of resilience may contribute to understanding the relationship between physical literacy and mental health in a sample of Chinese college students.

In accordance with the definition of mental health and the pathway from physical literacy to mental health (46), this study found that physical literacy was a significant predictor of mental health. Together, this indicates that college students who have higher physical literacy tend to enjoy greater mental health. This finding was consistent with one previous study, which showed positive relationship between physical literacy and positive affect among early adolescents (20). Such association proved to some extent that in addition to developing physical health, physical literacy is inextricably related to mind factors (3).

One potential mechanism linking physical literacy to enhanced metal health is through physical competence and associated perceptions of competence (i.e., confidence). Experiencing perceptions of competence is considered essential for psychological growth and wellness (47). Indeed, if the affective dimensions of physical literacy, such as *motivation and confidence*, go beyond just motor action, then they may also help to promote mental health, and support young adults to the pursuit of a harmony state between the health of the body and the mind. Another potential explanation for the relationship between physical literacy and mental health may be attributed to improved physical activity level. Physical literacy promoted physical activity levels (8, 9); physical activity contributed to positive mental health (48). Moreover, our results support our third hypothesis that resilience represents a potential underlying mechanism that could partially explain how physical literacy is linked with mental health. That is, promoting physical literacy as a way to build up resilience could help to improve mental health among college students. A positive relationship between physical literacy and resilience has been demonstrated in a previous study (23) and the current study. Physical literacy can be a good booster in the process of developing the resilience of college students. Emotional domains, including *motivation and confidence*, of physical literacy contributed to the fundamental to resilience, since they may provide or assist individuals acquire the skills and abilities to better negotiate for, and navigate to, resources that sustain their wellbeing in different circumstances (23). The positive challenge faced in the process of developing physical competence may also position the physical literacy as an antecedent of resilience (23). On the other hand, college students with a high level of resilience tend to have confidence in dealing with challenges and adversity, and to be able to cope with difficulties; they are more likely to evaluate their mental health with a positive attitude. Our results show no difference with those of previous studies that resilience has been regarded as the defense mechanism for people who are emotionally depressed after facing setbacks, and could promote mental health (49). Moreover, previous studies have brought the relationship between resilience and mental health to interdisciplinary field discussions and have conducted various models based on numerous theoretical and empirical studies (50). This finding was also in line with our results, where resilience is correlated with the psychological wellbeing dimension and shows a strong correlation with wellbeing at the social and emotional dimensions (33). Therefore, the contribution of physical literacy to health may be not only at the level of physical health but also at a more macro level, including both physical and mental health. The statement made by Whitehead, physical literacy should be considered as intrinsic to human flourishing (51, 52), also support these ideas. This is particularly important given the rising mental health issue reported among college students (53, 54). Our findings suggested that physical literacy may be an optimizing way through which these mental issues might be alleviated.

To better understand the mediator role of resilience among physical literacy and mental health, this study also explored the mediating effect of resilience on the relationship between physical literacy and each dimension of mental health, namely, emotional, psychological, and social wellbeing. Based on our model, resilience is the partial mediator in the model of psychological wellbeing. Specifically, resilience can strengthen or weaken the correlation of physical literacy for psychological wellbeing. Psychological wellbeing has been considered as a set of psychological features involved in positive human functioning under the “*eudaimonic* perspective” (13). Theory-guided dimensions of psychological wellbeing including self-acceptance (positive attitude toward the self), positive relations with others, autonomy (self-determining and independent), environmental mastery (has a sense of mastery and competence in managing environment), purpose in life (goals in life and sense of directedness), and personal growth (55). Physically literate individuals with high confidence, enthusiasm for life, and ability to interact with the environment, thus, tend to show high psychological wellbeing. Meanwhile, psychological well-being includes several resilience-related aspects, such as purpose in life (33). Indeed, resilience has been demonstrated to be the predictor of psychological wellbeing (56). Similar to this, resilience is the partial mediator between physical literacy and social wellbeing. Physical literacy improves the ability of individuals to interact with the social environment, and resilience in adversity continues to amplify such ability, thus, maximizing social wellbeing. In addition, it is worth noting that, according to our mediating model, resilience is the full mediator between physical literacy and emotional well-being. In other words, physical literacy improves emotional well-being entirely by promoting resilience. This has very important implications for practice. For example, in physical literacy curricula development, only by cooperating the importance of both physical and psychological factors related to resilience can such a holistic construction promote all three dimensions of mental health among college students.

Nowadays, mental disorders are becoming one of the major diseases in the world (57). College students, especially freshmen, were the majority of patients (58). In this respect, Chinese undergraduates showed no difference from the rest of the world (59). Mental health problems affect the academic performance and behavioral habits of students (60). In the long run, the mental health problem of students was considered as one of the primary obstacles to the continuation of higher education. Mental and psychological health have been recognized as important as physical health and need to be included in health and social policy considerations. In this context, this study provides a novel perspective, encouraging physical literacy to foster resilience and subsequently promote mental health, to address the issue. This has significant practical implications for curriculum development in the universities. Universities can reasonably offer a curriculum designed on the basis of physical literacy and resilience to improve the mental health of students. For example, adding more motivation and confidence building to the physical education course, or taking students to different environments to feel the changes in their bodies and to develop adaptive capacity. Not only physical education course but also other courses can integrate elements of resilience into their curriculum design, including setting relatively difficult and positively challenging content, and encouraging students to overcome them on their own. Students can also be encouraged to participate in meaningful extracurricular activities and try to adapt to different environments. In the process, students will feel psychological satisfaction, which will bring wellbeing and, thus, improve their quality of lives.

## Limitations

The present study was conducted with a group of Chinese undergraduates. Although the mental health issue of college students is a global problem, the different education systems among countries, including higher education, still have an impact on the mediated relationship. Given that University systems differ from country to country, the findings of this study showed limited generalization and applicability to countries with different education systems. Furthermore, the data in this article were self-reported. The measurements provided were not the observations of others, but rather an assessment of oneself. Each person will also have more or less different criteria for evaluation. This may result in deficiencies in the objectivity of this study. There are also limitations in some of the methodological decisions. The article discussed the three factors of mental health separately but did not consider each dimension of physical literacy as well. The different factors may have an impact on the results compared with a single whole. Future research could build a better model through structural equations to have a better explanation of the latent variables. Finally, although the results of this study support the hypothesized relationships described in the existing literature, any causal statements regarding the relationship between physical literacy, resilience, and mental health should be made with caution. Additional experimental studies are needed to verify the observed causal inferences.

## Conclusions

This study explores the relationship among physical literacy, mental health, and resilience, and directly links physical literacy with mind factors, making it a strong addition to the existing physical literacy research. Our findings support the development of physical literacy in universities as part of a holistic approach to support the wellbeing and mental health of undergraduates. This study provides a new perspective of intervention for improving mental health of college students. Physical education programs can play an important role in this process by designing programs that focus on the concept of physical literacy, thereby improving both the physical and mental health of students. At the same time, other courses can also incorporate resilience-based content into their curriculum to improve resilience of the students and, thus, their wellbeing.

## Data Availability Statement

The raw data supporting the conclusions of this article will be made available by the authors, without undue reservation.

## Ethics Statement

The studies involving human participants were reviewed and approved by IRB of Jinan University (JNUKY-2021-008). The patients/participants provided their written informed consent to participate in this study.

## Author Contributions

RM and TL were responsible for conceptualization, formal analysis, and writing. All authors were in charge of collecting and analyzing data and reviewed and approved the manuscript.

## Conflict of Interest

The authors declare that the research was conducted in the absence of any commercial or financial relationships that could be construed as a potential conflict of interest.

## Publisher's Note

All claims expressed in this article are solely those of the authors and do not necessarily represent those of their affiliated organizations, or those of the publisher, the editors and the reviewers. Any product that may be evaluated in this article, or claim that may be made by its manufacturer, is not guaranteed or endorsed by the publisher.

## References

[1] Whitehead M. Physical Literacy: Throughout the Lifecourse. London: Routledge (2010).

[2] IPLA. Home Page. (2017). Available online at: https://www.physical-literacy.org.uk (accessed August 31, 2021).

[3] Whitehead M. Physical Literacy Across the World. London: Routledge (2019).

[4] Cairney J Kiez T Roetert EP Kriellaars D. A 20th-century narrative on the origins of the physical literacy construct. J Teach Phys Educ. (2019) 38:79–83. 10.1123/jtpe.2018-0072

[5] Caldwell HAT Wilson A Mitchell D Timmons BW. Development of the Physical Literacy Environmental Assessment (PLEA) tool. PLoS ONE. (2020) 15:e0230447. 10.1371/journal.pone.0230447 32182272 PMC7077881

[6] Keegan R Keegan S Daley S Ordway C Edwards A. Getting Australia Moving: Establishing a Physically Literate Active Nation (Game Plan). Canberra, ACT: University of Canberra (2013).

[7] Spengler JO Cohen J. Physical Literacy: A Global Environmental Scan. Washington, DC: Aspen Institute Sports and Society Program (2015).

[8] Kwan MYW Graham JD Healey C Paolucci N Brown DM. Stopping the drop: examining the impact of a pilot physical literacy-based intervention program on physical activity behaviours and fitness during the transition into University. Int J Environ Res Public Health. (2020) 17:1–12. 10.3390/ijerph17165832 32806584 PMC7459702

[9] Ma RS Sum RKW Li MH Huang Y Niu XL. Association between physical literacy and physical activity: a multilevel analysis study among chinese undergraduates. Int J Environ Res Public Health. (2020) 17:1–12. 10.3390/ijerph17217874 33121068 PMC7663683

[10] Caldwell HAT Di Cristofaro NA Cairney J Bray SR Macdonald MJ Timmons BW. Physical literacy, physical activity, and health indicators in school-age children. Int J Environ Res Public Health. (2020) 17:1–12. 10.3390/ijerph17155367 32722472 PMC7432049

[11] WHO. Mental Health. (2021). Available online at: https://www.who.int/mental_health/who_urges_investment/en/ (accessed August 31, 2021).

[12] Deci EL. Ryan RM. Hedonia, eudaimonia, and well-being: an introduction. J Happiness Stud. (2008) 9:1–11. 10.1007/s10902-006-9018-1

[13] Ryff CD. Happiness is everything, or is it? explorations on the meaning of psychological well-being. J Pers Soc Psychol. (1989) 57:1069. 10.1037/0022-3514.57.6.106926400043

[14] Keyes CLM. Social well-being. Soc Psychol Q. (1998) 61:121–40. 10.2307/2787065

[15] Waterman AS. Two conceptions of happiness: contrasts of personal expressiveness (eudaimonia) and hedonic enjoyment. J Pers Soc Psychol. (1993) 64:678–91. 10.1037/0022-3514.64.4.678

[16] WHO. Mental Health: Strengthening Our Response. (2021). Available online at: https://www.who.int/news-room/fact-sheets/detail/mental-health-strengthening-our-response (accessed August 31, 2021).

[17] Espie CA Emsley R Kyle SD Gordon C Drake CL Siriwardena AN. Effect of digital cognitive behavioral therapy for insomnia on health, psychological well-being, and sleep-related quality of life: a randomized clinical trial. JAMA Psychiatry. (2019) 76:21–30. 10.1001/jamapsychiatry.2018.2745 30264137 PMC6583463

[18] Almond L. Physical literacy and fundamental movement skills: an introductory critique. ICSSPE Bull J Sport Sci Phys Educ. (2013) 65:80–8. Retrieved from: https://www.icsspe.org/sites/default/files/bulletin65_0.pdf

[19] Wang L Zhou Y Wang F Ding L Love PE Li S. The influence of the built environment on people's mental health: an empirical classification of causal factors. Sustain Cities Soc. (2021) 74:103185. 10.1016/j.scs.2021.103185

[20] Blain DO Curran T Standage M. Psychological and behavioral correlates of early adolescents' physical literacy. J Teach Phy Educ. (2020) 40:157–65. 10.1123/jtpe.2019-0131

[21] Wang FJ Cheng CF Chen MY Sum KWR. Temporal precedence of physical literacy and basic psychological needs satisfaction: a cross-lagged longitudinal analysis of University students. Int J Environ Res Public Health. (2020) 17:4615. 10.3390/ijerph17124615 32604980 PMC7345862

[22] Masten AS. Global perspectives on resilience in children and youth. Child Dev. (2014) 85:6–20. 10.1111/cdev.12205 24341286

[23] Jefferies P Ungar M Aubertin P Kriellaars D. Physical literacy and resilience in children and youth. Front Public Health. (2019) 7:346. 10.3389/fpubh.2019.00346 31803709 PMC6877541

[24] Ungar M Ghazinour M Richter J. Annual research review: what is resilience within the social ecology of human development? J Child Psychol Psychiatry Allied Discip. (2013) 54:348–66. 10.1111/jcpp.12025 23215898

[25] Dudley D Cairney J Wainwright N Kriellaars D Mitchell D. Critical considerations for physical literacy policy in public health, recreation, sport, and education agencies. Quest. (2017) 69:436–52. 10.1080/00336297.2016.1268967

[26] Rutter M. Resilience as a dynamic concept. Dev Psychopathol. (2012) 24:335–44. 10.1017/S0954579412000028 22559117

[27] Brewer ML Van KG Sanderson B Naumann F Lane M Reubenson A. Resilience in higher education students: a scoping review. High Educ Res Dev. (2019) 38:1105–20. 10.1080/07294360.2019.162681031706205

[28] Durlak JA Weissberg RP Dymnicki AB Taylor RD Schellinger KB. The impact of enhancing students' social and emotional learning: a meta-analysis of school-based universal interventions. Child Dev. (2011) 82:405–32. 10.1111/j.1467-8624.2010.01564.x 21291449

[29] Schussler DL Greenberg M DeWeese A Rasheed D DeMauro A Jennings PA. Stress and release: case studies of teacher resilience following a mindfulness-based intervention. Am J Educ. (2018) 125:1–28. 10.1086/699808

[30] Mampane R Huddle C. Assessing the outcomes of school-based partnership resilience intervention. S Afr J Educ. (2017) 37:1–13. 10.15700/saje.v37n1a1412

[31] Challen A Noden P West A Machin S. UK Resilience Programme Evaluation: Final Report REPORT. (2014). Available online at: http://eprints.lse.ac.uk/51617/ (accessed August 31, 2021).

[32] Pan-Canadian Joint Consortium for School Health. JCSH Positive Mental Health Toolkit. Available online at: http://www.jcsh-cces.ca/ (accessed August 31, 2021).

[33] Sagone E Caroli ME De. Relationships between psychological Well-being and resilience in middle and late adolescents. Proc Soc Behav Sci. (2014) 141:881–7. 10.1016/j.sbspro.2014.05.154

[34] Moore THM Kesten JM López-López JA Ijaz S McAleenan A Richards A. The effects of changes to the built environment on the mental health and well-being of adults: systematic review. Health Place. (2018) 53:237–57. 10.1016/j.healthplace.2018.07.012 30196042

[35] Zhao X Fu F Zhou L. The mediating mechanism between psychological resilience and mental health among left-behind children in China. Child Youth Serv Rev. (2020) 110:104686. 10.1016/j.childyouth.2019.104686

[36] Ng R Ang RP Ho MHR. Coping with anxiety, depression, anger and aggression: the mediational role of resilience in adolescents. Child Youth Care Forum. (2012) 41:529–46. 10.1007/s10566-012-9182-x

[37] Yildirim M Arslan G Özaslan A. Perceived risk and mental health problems among healthcare professionals during COVID-19 pandemic: exploring the mediating effects of resilience and coronavirus fear. Int J Ment Health Addict. (2020) 1–11. 10.1007/s11469-020-00424-8 33223977 PMC7668285

[38] Nath P Pradhan RK. Influence of positive affect on physical health and psychological well-being: examining the mediating role of psychological resilience. J Health Manag. (2012) 14:161–74. 10.1177/097206341201400206

[39] Whitehead M. Definition of physical literacy and clarification of related issues. ICSSPE Bull. (2013) 65:28–33. Retrieved from: https://www.icsspe.org/sites/default/files/bulletin65_0.pdf

[40] Regehr C Glancy D. Pitts A. Interventions to reduce stress in University students: a review and meta-analysis. J Affect Disord. (2013) 148:1–11. 10.1016/j.jad.2012.11.026 23246209

[41] Ma RS Sum RKW Hu YN Gao TY. Assessing factor structure of the simplified Chinese version of Perceived Physical Literacy Instrument for undergraduates in Mainland China. J Exerc Sci Fit. (2020) 18:68–73. 10.1016/j.jesf.2020.01.001 31998384 PMC6965736

[42] Guo C Tomson G Guo J Li X Keller C Söderqvist F. Psychometric evaluation of the Mental Health Continuum-Short Form (MHC-SF) in Chinese adolescents–a methodological study. Health Qual. Life Outcomes. (2015) 13:1–9. 10.1186/s12955-015-0394-2 26651829 PMC4676120

[43] Mu GM Hu Y. Validation of the Chinese version of the 12-item child and youth resilience measure. Child Youth Serv Rev. (2016) 70:332–9. 10.1016/j.childyouth.2016.09.037

[44] Ungar M Liebenberg L. Assessing resilience across cultures using mixed methods: construction of the Child and youth resilience measure. J Mix Methods Res. (2011) 5:126–49. 10.1177/1558689811400607

[45] Rockwood NJ Hayes AF. MLmed: An SPSS Macro for Multilevel Mediation Conditional Process Analysis. (2017). Available online at: www.afhayes.com (accessed August 31, 2021).

[46] HMG/DH. No Health Without Mental Health: A Cross-Government Mental Health Outcomes Strategy for People Of All Ages. London: Dep Health (2011).

[47] Ryan RM Deci EL. Self-Determination Theory: Basic Psychological Needs in Motivation, Development, and Wellness. New York, NY: The Guilford Press (2017).

[48] Guo C Tomson G Keller C Söderqvist F. Prevalence and correlates of positive mental health in Chinese adolescents. BMC Public Health. (2018) 18:1–11. 10.1186/s12889-018-5133-2 29454315 PMC5816379

[49] Davydov DM Stewart R Ritchie K Chaudieu I. Resilience and mental health. Clin Psychol Rev. (2010) 30:479–95. 10.1016/j.cpr.2010.03.003 20395025

[50] Hu T Zhang D Wang J A. meta-analysis of the trait resilience and mental health. Pers Individ Dif. (2015) 76:18–27. 10.1016/j.paid.2014.11.039

[51] Durden-Myers EJ Whitehead ME Pot N. Physical literacy and human flourishing. J Teach Phys Educ. (2018) 37:308–11. 10.1123/jtpe.2018-0132

[52] Robinson DB Durden-Myers EJ Berg S. Physical literacy and flourishing (within) Canadian school communities. In: Cherkowski S Walker K editors. Perspectives on Flourishing Schools. Lanham, MD: Lexington Books (2018). p. 199.

[53] Coiro MJ Bettis AH Compas BE. College students coping with interpersonal stress: examining a control-based model of coping. J Am Coll Health. (2017) 65:177–86. 10.1080/07448481.2016.1266641 27911672

[54] Mustafa RM Alrabadi NN Alshali RZ Khader YS Ahmad DM. Knowledge, attitude, behavior, and stress related to covid-19 among undergraduate health care students in Jordan. Eur J Dent. (2020) 14:S50–5 10.1055/s-0040-1719212 33233003 PMC7775211

[55] Ryff CD Singer B. Psychological well-being: meaning, measurement, and implications for psychotherapy research. Psychother Psychosom. (1996) 65:14–23. 10.1159/000289026 8838692

[56] Souri H Hasanirad T. Relationship between resilience, optimism and psychological well-being in students of medicine. Procedia Soc Behav Sci. (2011) 30:1541–4. 10.1016/j.sbspro.2011.10.299

[57] Sowers KM Dulmus CN Linn BK. Mental Illness: Worldwide. Encyclopedia of Social Work. Oxford: NASW Press and Oxford University Press (2019).

[58] Bruffaerts R Mortier P Kiekens G Auerbach RP Cuijpers P Demyttenaere K. Mental health problems in college freshmen: prevalence and academic functioning. J Affect Disord. (2018) 225:97–103. 10.1016/j.jad.2017.07.044 28802728 PMC5846318

[59] Liu F Zhou N Cao H Fang XY Deng LY Chen WR. Chinese college freshmen's mental health problems and their subsequent help-seeking behaviors: a cohort design (2005–2011). PLoS ONE. (2017) 12:e0185531. 10.1371/journal.pone.0185531 29040266 PMC5644985

[60] Grøtan K Sund ER Bjerkeset O. Mental health, academic self-efficacy and study progress among college students–The SHoT study, Norway. Front Psychol. (2019) 10:45. 10.3389/fpsyg.2019.00045 30733694 PMC6354661

